# Smart Textiles for Visible and IR Camouflage Application: State-of-the-Art and Microfabrication Path Forward

**DOI:** 10.3390/mi12070773

**Published:** 2021-06-30

**Authors:** Lauren M. Degenstein, Dan Sameoto, James D. Hogan, Asad Asad, Patricia I. Dolez

**Affiliations:** 1 Department of Human Ecology, University of Alberta, Edmonton, AB T6G 2N1, Canada; degenste@ualberta.ca; 2 Department of Mechanical Engineering, University of Alberta, Edmonton, AB T6G 2N1, Canada; sameoto@ualberta.ca (D.S.); jdhogan@ualberta.ca (J.D.H.); aaasad@ualberta.ca (A.A.)

**Keywords:** camouflage, visible and infrared (IR) signature, protective clothing, smart textiles, microfabrication

## Abstract

Protective textiles used for military applications must fulfill a variety of functional requirements, including durability, resistance to environmental conditions and ballistic threats, all while being comfortable and lightweight. In addition, these textiles must provide camouflage and concealment under various environmental conditions and, thus, a range of wavelengths on the electromagnetic spectrum. Similar requirements may exist for other applications, for instance hunting. With improvements in infrared sensing technology, the focus of protective textile research and development has shifted solely from providing visible camouflage to providing camouflage in the infrared (IR) region. Smart textiles, which can monitor and react to the textile wearer or environmental stimuli, have been applied to protective textiles to improve camouflage in the IR spectral range. This study presents a review of current smart textile technologies for visible and IR signature control of protective textiles, including coloration techniques, chromic materials, conductive polymers, and phase change materials. We propose novel fabrication technology combinations using various microfabrication techniques (e.g., three-dimensional (3D) printing; microfluidics; machine learning) to improve the visible and IR signature management of protective textiles and discuss possible challenges in terms of compatibility with the different textile performance requirements.

## 1. Introduction

Today’s protective textiles must defend against a multitude of threats and fulfill a variety of functional requirements. In particular, textiles for military applications require durability, resistance to ballistic threats and environmental conditions (e.g., ultraviolet (UV) light, moisture, fire, heat, and wind), all while being comfortable and lightweight [1]. In addition, these textiles must provide camouflage and concealment under various environmental conditions and, thus, a range of wavelengths on the electromagnetic spectrum, especially the visible region (400–800 nm), near-infrared region (NIR) (750–1200 nm), and thermal or far-infrared region (FIR) (3–5 and 8–14 μm) [2]. Textiles must also adhere to military textile standard specifications/requirements such as colorfastness to light, washing, and perspiration to ensure changes to visible or infrared concealment are not compromised in use or as a result of cleaning [3].

Historically, military textiles for close-range battle were colorful and bright, intended for enemy intimidation and regimental identification [2]. With advancements in long-range weaponry and visual detection equipment at the beginning of the 20th century, the purpose of military uniforms was now to blend into the soldier’s background [2]. Although the desired colors may differ based on the wearer’s environment, modern camouflage textiles are typically olive, green, khaki, brown and black [3]. 

Camouflage properties for military textiles can come in the form of clothing, light flexible nets, garnishing and covers [2]. Textiles are flexible, three-dimensional materials made from fibers that can be spun into yarns and woven (interlaced) or knitted (interlooped) into a fabric. Alternatively, non-woven textiles can be made by bonding fibrous webs through mechanical entanglement, using resin, or thermally or chemically fusing the fibers together [4]. Embroidery is also used for the creation of technical textiles where yarns or threads are stitched on to the surface of a ground material [4]. Additional functional and aesthetic properties can be imparted into a textile by the application of surface finishes, coatings, or lamination techniques. Military textiles have traditionally been made from woven cotton fabrics, eventually blending with synthetic fibers such as nylon to be more lightweight and to dry more quickly [5]. As fiber manufacturing technology improved, the addition of high-performance fibers such as para-aramids and ultra-high molecular weight polyethylene (UHMWPE) have been integrated into uniforms for ballistic protection [6]. Protective fabrics are continuously being improved as new threats arise and technologies to address these threats are integrated into textile systems.

The diverse requirements of military fabrics have been a motivator for the rapid development of smart textiles [7,8]. Put simply, smart or intelligent textiles are textiles that can automatically sense, react, and adapt to environmental stimuli [7]. There are varying degrees of “smartness” within a textile system [9] (p. 109). At a passive level, textiles will only sense an external stimulus; active smart textiles have an added sensor and actuator or display function; and responsive or ultra-intelligent textiles sense, react, and adapt to stimuli [9,10]. A simple smart textile can automatically sense and react to a stimulus as with responsive phase change, chromatic or shape memory materials or an active sensor can be added to the material to detect external stimuli [9,11]. This sensor may respond to the stimuli in a manner that is directly visible (e.g., changes to physical properties such as color, shape, or size), through an indirect response at the molecular level, or by electric or magnetic mechanisms [9]. These responses may be undetectable to the naked eye, but still trigger a controlled reaction [9]. 

Smart technologies can be integrated into fabrics to protect against various threats including biological, mechanical, or chemical hazards, or to improve the functionality of fabrics such as changing appearance or through enhanced thermal regulation. These technologies are applied through different techniques and mediums such as nanoparticles, microencapsulation, lamination, woven or knitted into a textile, or added at the polymer stage prior to fiber extrusion. There are three generations of smart textiles, which are differentiated based on the stage of the manufacturing process they are added [10,12]. First generation smart textiles are added to a fully assembled garment; for second generation textiles, the active component is incorporated during textile manufacturing such as weaving or knitting; lastly, active components are integrated within the fiber or yarns of a textile for third generation smart textiles [10,12]. While the possibilities for smart textile applications are diverse, the functionality of smart textiles must be compatible with how these textiles are used and maintained in its real-world application. Therefore, it is important to consider how smart solutions may interfere with textile performance.

In this article, we review various smart textile mechanisms from simple to increasing complexity and how they have been applied for the purpose of achieving visible and infrared (IR) camouflage. Section 2 describes these existing technologies applicable to visible and IR camouflage for textiles. Following this, Section 3 reports on current progress in terms of adaptive camouflage. Lastly, Section 4 discusses the requirements and potential strategies for improved visible and IR signature management using microfabrication techniques.

## 2. Existing Technologies Applicable to Visible and IR Camouflage

Various techniques with the potential to develop smart camouflage textiles have been identified and are described in detail in this section. They involve different levels of responsiveness to control the visible and/or signature of objects. Table 1 gives an overview of their advantages and current limitations for application in smart camouflage textiles, which will be discussed in greater detail in Section 4.

### 2.1. Surface Dyeing and Pigmentation

**Figure 1:**
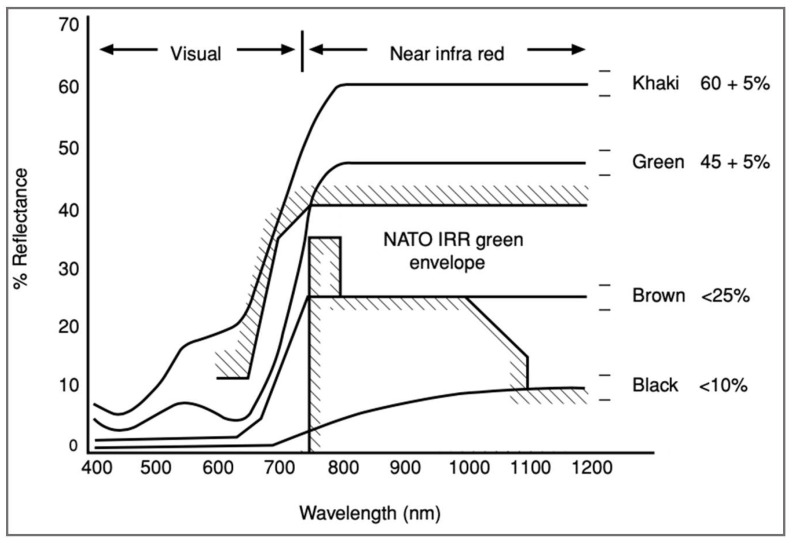
Spectral reflectance curves of four colors commonly used for military camouflage textiles in the visual and near infrared wavelengths. The North Atlantic Treaty Organization (NATO) near-infrared green envelope has been superimposed over the reflectance curves (reproduced from [2] with permission from Elsevier).

Visible camouflage in military fabrics is generally achieved using colors and patterns that resemble the color, intensity, pattern, texture, and appearance of a soldier’s natural or artificial environment through dyeing or pigmentation techniques [2,21]. Dyed or pigmented fabrics’ CIE (Commission International de l’Eclairage) color parameters are measured using spectrophotometers, which identify colors based on its lightness, red/green and yellow/blue values, and its reflectance properties [22,23]. Textiles also must be colored using NIR reflecting dyes or pigments to match the reflectance curves or “chlorophyll rise” of vegetation in the NIR range [2,21] (p. 444). Spectral reflectance curves for the colors often associated with camouflage range from <10% (black), <25% (brown), 45 ± 5% (green) and 60 ± 5% for light green/khaki colorants (Figure 1) [2,24]. While tolerance levels for reflectance values to match the colors of natural objects may be specified by military authorities, specific dye synthesis, formulations, and applications have remained mostly confidential due to the sensitive nature of IR camouflage research [3].

Ease of dyeing depends on the absorbency of the fiber as well as availability of reactive sites in the fiber’s molecular structure [4], indicating that different fibers require different dye classes. High-performance fibers (e.g., polyethylene, polypropylene, polytetrafluoroethylene) are difficult to dye due to the fibers’ hydrophobicity, high crystallinity, and limited functional groups to act as dye sites [13]. These fibers can be colored either by adding pigment at the fiber formation stage (mass pigmentation), by chemically modifying the fiber in the dyeing process [25] or printed using pigments or dyes. Cotton fibers are most often vat dyed or pigment printed using anthraquinone type dyes to achieve NIR reflectance [21], while few conventional dyes for wool and synthetic fibers have sufficient infrared absorption [3]. Despite potential dyeing challenges, NIR reflectance matching has been achieved by dyeing cotton fabric with vat dyes [26,27] and dyeing poly(ethylene terephthalate) PET or PET/cotton blended fabrics with disperse dyes [28] to mimic the NIR reflectance curves of greenish deciduous and coniferous leaves. 

Rubeziene et al. studied the visible and NIR camouflage of various dye combinations (pigment printing; reactive and disperse dyes; vat and disperse dyes) printed on four cotton/polyester blended fabrics, before and after various fabric tests [22]. These tests included an assessment of the fabrics’ color change after abrasion, flexing, washing and light exposure, and demonstrated that repeated washing and light exposure can significantly impact color change and therefore camouflage ability, while abrasion and flexing color change was inconsiderable. They achieved the best results in terms of visual color change after 20 wash cycles and 50 h of light exposure with a 50% cotton/50% polyester fabric printed with reactive and disperse dyes, although most fabrics remained within the acceptable NIR range after these tests. 

In addition to using NIR reflecting dyes and pigments, other techniques for providing IR camouflage include the incorporation of IR absorbing pigments in the printing paste and the fiber polymer itself, as well as finishes that minimize the fabric’s IR reflectance [22,26]. In their study applying various inorganic pigment formulations to cotton and nylon fabrics, Gupta et al. found that specific pigments with particularly high or low IR reflectance values (e.g., carbon black, sudarshan yellow, and signal red) had a significant effect on the overall IR reflectance value of the printed fabric [29]. The authors state that adding or removing these pigments, even in small quantities, could significantly change the formulation’s IR reflectance values, where the addition of carbon black lowers the resultant IR reflectance values while adding sudarshan yellow and signal red raises them [29]. The study found that, with all other constituents being constant, increasing the binder content of the pigment formulation also slightly raised the IR reflectance value. Additionally, they compared the reflectance values of cotton and nylon with the same applied pigment formulations and binder content and found that nylon had higher reflectance values due to the fiber’s cross-sectional (rounded) and longitudinal shape (smoother and more uniform). This suggests that the fiber type will also influence the IR reflectance values of a fabric due to the way light reflects off various fiber shapes. 

NIR reflectance can also be modified through the incorporation of micro or nanoparticle additives at the fiber manufacturing stage [26] or printing processes. Simply adding nanoparticles that can tune the reflectance spectra of pigment shades can be a cost-effective solution without increasing the amount of dyes and pigments [30]. Inorganic materials such as metals, metal oxides and metal compounds are often chosen because they do not absorb radiation in the mid-IR spectrum as with traditional organic dyes and are, thus, inconsequential to the IR transparency of the composite even in increased concentrations [31,32]. Salehi et al. investigated the visible and NIR camouflage of viscose/polyester fabrics printed with two mineral pigments (carbon black (CB) and activated carbon nanoparticles) added to green and black vat and disperse printing pastes [23]. The addition of the CB and activated carbon nanoparticles significantly reduced the fabric’s IR reflectance for both green and black samples. The IR reflectance of green samples was reduced to <20%, making it below the accepted NATO range and therefore identifiable at such a low reflectance. However, the added CB and activated carbon nanoparticles reduced the IR reflectance of the black sample to an acceptable range (<10%). Their findings suggest that the addition of CB and activated carbon nanoparticles may be advantageous for some colors (e.g., black), but not necessary for other colors that can achieve the acceptable IR reflectance range from suitable dyes or printing pastes alone (e.g., green). The researchers also examined the color fastness, water absorption time, air permeability, strength, bending length and crease recovery of the black and green samples. They found that the added nanoparticles decreased the fabric sample’s air permeability, strength and bending length, demonstrating the potential trade-offs between IR and mechanical performance in these applications. 

Care must also be taken that added nanoparticles do not significantly alter the visual color of the textile. Khajeh Mehrizi et al. printed cotton/nylon fabrics with varying amounts of CB nanoparticles (0.05–0.50 g/kg of CB in printing paste) and pigments to tune the fabrics’ visible and NIR camouflage for a desert environment [30]. Increased CB nanoparticles had a greater impact on reflectance values (increased NIR absorption) for lighter colors such as olive green and khaki that initially had higher reflectance curves than it did for darker colors such as brown with lower initial reflectance curves. The addition of CB nanoparticles also caused a noticeable color difference between the original reference samples and the treated samples. Consequently, the authors suggest adding a low concentration of CB nanoparticles (0.05 g/kg of printing paste) for visible and NIR concealment. Similar results were achieved when Khajeh Mehrizi et al. added multiwalled carbon nanotube particles (MWCNTs) with a concentration range of 0.04–0.12 g kg^−1^ to the printing paste applied to 50% cotton/50% nylon fabric [14]. The addition of MWCNTs lowered the NIR reflectance values of the dark brown, light brown and olive printed fabrics while also increasing the visible reflectance of the samples. Again, the authors suggest adding low concentrations of MWCNTs to the printing paste for both IR and visible reflectance of the fabrics.

Another study by Abbasipour and Khajeh Mehrizi investigated the reflectance behavior of 65% cotton/35% polyester fabrics printed using pigment or vat dye printing pastes with added anatase titanium dioxide (TiO_2_) or CB powder [15]. Standard samples of printed fabrics were compared to samples with either 0.01 or 0.05 g/kg of TiO_2_ or CB concentration in the printing paste. Interestingly, the authors found that the reflectance percentages of the samples printed with vat dyes and the addition of TiO_2_ and CB did not differ from the standard samples, while the pigment printed samples had a decreased reflectance in the NIR region with the added particles. Again, there were significant visual color differences with the addition of TiO_2_ and CB compared to the standard, with increased concentrations causing greater color differences for most of the samples. All samples had good fastness to light, rubbing and washing, indicating adequate fiber and pigment adhesion. 

Similarly, Siadat and Mokhtari coated printed cotton/nylon fabrics in woodland and desert patterns with varying concentrations (1–2% *w*/*v* (%)) of zirconium dioxide (ZrO_2_) and cerium dioxide (CeO_2_) nanoparticles with and without a 6% (*w*/*v* (%)) citric acid cross-linker using a pad-dry-cure process [16]. They found that when the concentration of the nanoparticle coating was at 2%, a CIE calculated color difference greater than 2 was found in all samples when compared to the uncoated printed fabrics (standard sample) and was therefore considered outside of the desired performance range due to a noticeable visual color difference. Therefore, the authors concluded that a 1% concentration of ZrO_2_ with 6% citric acid coating applied to the cotton/nylon fabrics was suitable for imitating the reflectance of woodland and desert environments in the NIR and SWIR regions, had an acceptable visual color difference compared to the standard fabric sample, and performed well in washing and rubbing fastness tests with ratings higher than 4 out of 5 for rubbing fastness. In a separate study, Siadat and Mokhtari coated jungle and desert pattern printed cotton/nylon fabrics in a similar manner, this time with nano ZrO_2_ and magnesium oxide (MgO) [32]. The padding solution contained 0.5%, 1%, 1.5%, and 2% (*w*/*v* (%)) of the metal oxide nanoparticles and a citric acid crosslinking agent of 6% (*w*/*v* (%)). This study determined that 1.5% ZrO_2_ and MgO nanoparticles were effective in decreasing reflectance levels over NIR and SWIR regions to match the jungle environment, with a further decrease in reflectance when the citric acid cross-linker was added. 

Samolov et al. impregnated camouflage printed cotton fabric with poly(vinyl butyral) (PVB) with and without fullerene-like tungsten disulfide (IF-WS_2_) nanoparticles [33]. The authors found that the addition of the nanoparticles enhanced the camouflage properties of the cotton fabric by lowering the diffuse reflectance of dark green and brown shades to an acceptable range according to the Serbian Military Standard.

Although the aforementioned coloration techniques are not necessarily considered “smart”, they have a higher level of functionality than consumer garments. Smart technologies could integrate these established dyeing and pigmentation techniques to improve visual and infrared camouflage ability of textiles.

### 2.2. Embedded Additives

**Figure 2:**
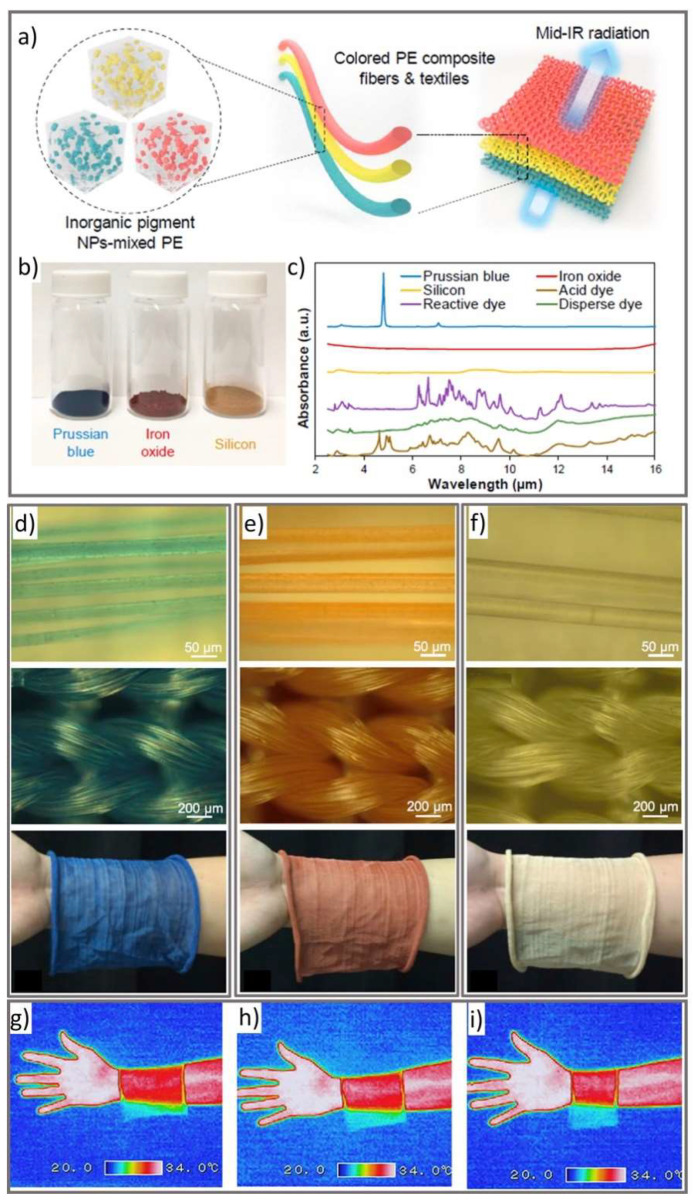
(**a**) Schematic illustration of mixing IR transparent nanosized pigments, Prussian blue, iron oxide (red) and silicon (yellow) with polyethylene material. The nanocomposite mixture was extruded as continuous fibers and used to make IR transparent knitted fabrics. (**b**) Optical image of the selected pigments. (**c**) The absorbance spectrum of the three pigments was analyzed using Fourier-transform infrared spectroscopy (FTIR) over the scanning range of 2–16 µm and compared with other traditional organic dyes. (**d**–**f**) show optical images of the extruded fibers, knitting patterns, and the final knitted textile of the three pigments. (**g**–**i**) show the IR images of the nanocomposite fabrics (reprinted from [31] with permission from Elsevier).

Colorants made of micro or nanoparticles can also be added at the polymer stage before fiber formation. Cai et al. mixed IR-transparent inorganic nanoparticle pigments (Prussian blue, iron oxide and silicon) with melted polyethylene pellets at a mass ratio of 1:100 before fiber extrusion [31]. These three pigments represent primary colors which can be used to make other colors by mixing them at varying ratios. The nanoparticle/polyethylene composite was extruded as a multi-filament yarn, which was then knitted into a fabric. The fabrics demonstrated ~80% IR transparency (Figure 2), a 1.6–1.8 °C passive cooling effect, as well as acceptable tensile strength and washability.

Esmaeilian et al. reported on the manufacture of PET filament yarns via low-speed melt-spinning with different concentrations of color index (C.I.) Pigment Green 7, C.I. Disperse Orange 123, and CB for camouflage in a forest environment [34]. PET granules were added to an extruder barrel, along with the dyes and CB particles, then extruded, drawn (stretched), and wrapped around spindles to form filament yarns. They found the optimum concentration to be C.I. Pigment Green 7 (0.1%), C.I. Disperse Orange 149 (0.05%), and inorganic CB (0.01%) to achieve an olive green hue. The addition of the green pigment reduced the NIR reflectance of the yarn by 15%. While the tenacity and elongation at break of the yarns with dyes and CB were slightly lower than the standard PET yarn, the mechanical properties of these yarns were still within the acceptable range. Furthermore, because the dyes and CB particles were embedded within the PET fibers, the yarns had excellent washing and light fastness.

In a subsequent study, Tavanaie et al. [35] used the same optimum concentration of C.I. Pigment Green 7 (0.1%) and inorganic CB (0.01%) as Esmaeilian et al. [34] in the production of camouflage PET yarns. However, they used a high-speed melt spinning process and C.I. Pigment Yellow 184 (0.05%) in place of the C.I. Disperse Orange 149. This time, the partially oriented PET filament yarns were drawn, texturized, and knitted into a fabric to investigate its reflectance properties. Tavanaie et al. concluded that the reflectance of the knitted fabric as measured by reflectance spectrophotometer simulated the olive reflectance spectra [35]. However, when examined using a thermal imager, the knitted fabric did not effectively simulate the ideal camouflage of the olive leaf reference due to differences in their respective pigment structures. To examine the effect of yarn geometry on reflectance properties, Kalan et al. compared the mass dyed multifilament yarns from their previous study [35] using simultaneous or conventional texturizing methods [36]. They found that yarn reflectance increased when the crimp per unit length of the yarn increased as the yarn became more compact and less bulky. As simultaneous texturizing caused greater yarn crimp and decreased mechanical properties, Kalan et al. determined that conventional texturizing methods were preferred for simulating olive hues in the NIR spectral region [36]. 

The use of conductive materials as additives can also enable the transfer of energy or information to carry out specific functions [9]. These conductive materials can be introduced at the solvent or polymer stage of fiber manufacturing, coated onto fabrics, or embedded within fibers, yarns, or fabrics [9]. Lim et al. developed a temperature-actuated conductive far-infrared (FIR) fiber that responds to changes in ambient temperature. In this study, a solution composed of polyurethane, dimethylformamide, tetrahydrofuran, tin (II) chloride dihydrate, and multiwalled carbon nanotubes was mixed and wet-spun in a coagulation bath [37]. The extruded fibers were dried and then dipped in a solution of phosphonic acid in isopropyl alcohol for hydrophobicity. By applying a voltage to the FIR fibers, the person or object emits the same IR waves as the background, effectively camouflaging the wearer. The IR emission of the FIR fibers could be tuned by adjusting the applied voltage or by controlling the electrical resistance of the fibers by increasing the carbon nanotube concentration.

In another study, Yu et al. designed a sheath-core bicomponent fiber with radar-absorbing materials [38]. The fibers were coextruded by melt spinning polypropylene chips with radar-absorbing materials including barium hexaferrite, manganese-zinc cubic ferrite and bronze powder in the core of the fiber and aluminum nanoparticles in the sheath. The addition of the aluminum nanoparticles did not affect the radar-absorbing properties of the fiber and reduced the infrared emissivity of the fiber to 0.62 at 15 wt.%, demonstrating the potential of this fiber as a suitable camouflage material.

### 2.3. Chromic Materials

Research for military applications has turned to the development of materials with coloration properties that can be tuned across the visible, IR and UV spectrum [7]. Textile colorants can be made tunable or react to external stimuli and radiate, change, or erase the color of a dyed, printed or finished fabric [7,9]. Stimuli can include changes in pH (ionochromism), light (photochromism), heat (thermochromism), moisture (solvation chromism), chemical reactions (chemichromism), electric or magnetic fields (electrochromism), or by applied stress or pressure (mechanochromism or piezochromism) [7,9].

**Figure 3:**
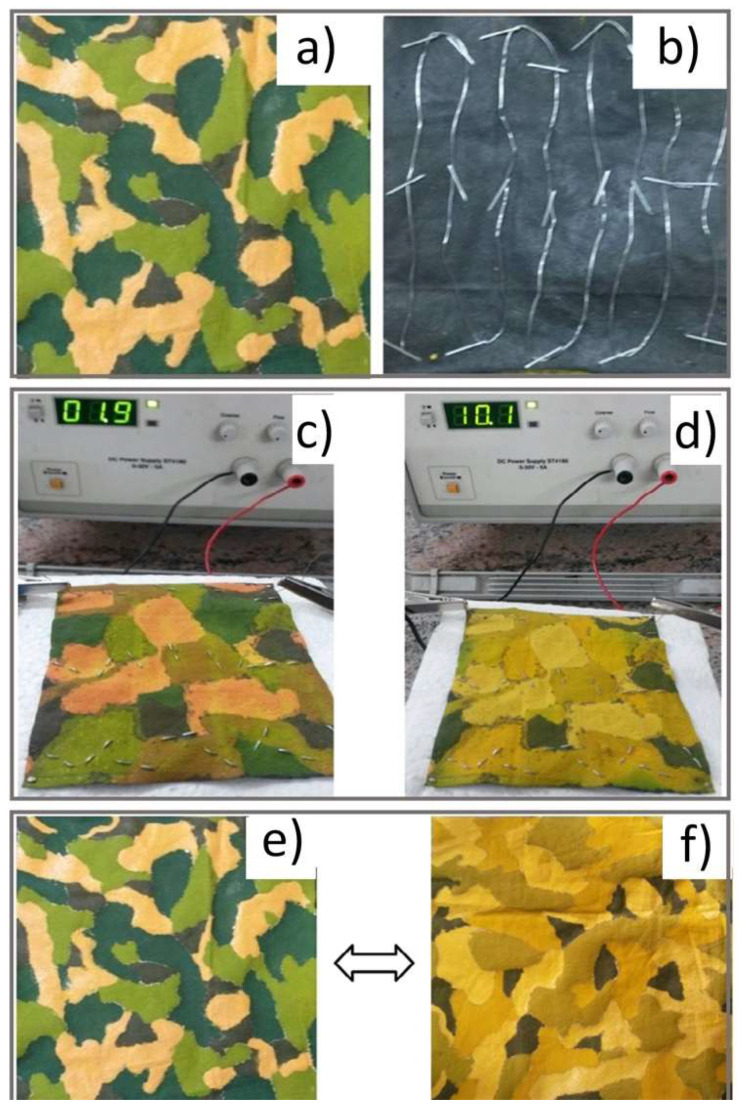
(**a**) The active side of camouflage fabric printed using thermochromic colorants. (**b**) The back side of the camouflage fabric coated with thermoplastic polyurethane layer containing liquid exfoliated graphite. The back side was also stitched with nichrome wires. (**c**,**d**) The change in color under applying 2 and 10 voltages, respectively. (**e**,**f**) The change in color of the camouflage patterns with and without heating at 60 °C for 2 minutes (reprinted from [39], ©The Textile Institute, with permission of Informa UK Limited, trading as Taylor & Francis Group, on behalf of The Textile Institute).

Karpagam et al. developed a camouflage printed fabric in a jungle motif using thermochromic colorants that exhibited a reversible color change with applied temperature and electrical power (Figure 3) [39]. Blue and orange thermochromic colorants, along with turmeric and graphite, were used to produce the printing paste for the jungle motif on the front of the cotton fabric. The back of the fabric was coated with thermoplastic polyurethane and liquid exfoliated graphene to improve conductivity and then stitched with a nichrome wire connected to a power supply. The color changing abilities of the coated fabric were tested by placing the sample in a hot air oven or by applying different voltages and currents to deliver heat. 

The researchers found that the time for a color change to occur varied depending on the color; for example, at 40 °C, brown changed to a lemon green color after 2 seconds, while dark green took 334 seconds to change to a light green [39]. All colors changed at a faster rate with increased temperature. Similarly, the colors changed more quickly as the voltage and current applied to the fabric increased. No color change was observed for any color at 1 V and 100 mA after 30 minutes, while all colors changed after 16 seconds at 7 V and 570 mA and reverted to their original colors after 5 minutes. Overall, the colors changed more quickly when voltage was applied compared to the oven heating method. Although the printed and coated fabric had suitable washing fastness, the fabric became stiffer, and its tensile and tear strength decreased from the printing process. The authors suggest finishing the fabric with an appropriate softener to prevent these functional shortcomings.

Viková and Pechová examined the camouflage properties of thermochromic inks on two different fabrics, one a plain weave cotton/polyester blend and the other a cotton/polyester blend with a Czech desert camouflage pattern [40]. Varying concentrations (10–600 g.kg^−1^) of thermochromic inks with transition temperatures ranging between 30 and 39 °C were dissolved with a dye precursor and color developer in a hydrophobic organic solvent, then encapsulated. These microcapsules were then mixed with a complex thickener consisting of water, glycerin, defoamer, binders, ammonia, and thickening agent before being screen printed onto the fabrics. A UV absorber and hydrophobic treatment were coated on the fabric to improve ink fastness. The measured thermochromic transition temperatures of the tested inks ranged from 30.7 ± 0.1 °C (green) to 38.6 ± 0.2 °C (black) with higher temperatures increasing the spectral reflectance of the camouflage fabrics. Ink fastness decreased when concentrations were above 300 g.kg^−1^ as the microcapsules were more easily rubbed off. However, the UV absorber and hydrophobic treatment helped to improve the light and washing fastness of the ink. Further work is needed to refine a tunable system with thermochromic inks.

### 2.4. Low Emissivity Coatings

In the far-infrared or thermal region of the electromagnetic spectrum (3–5 μm and 8–14 μm), objects can be detected by their emitted or reflected heat energy [2]. Within these bands, the atmosphere is transparent enough to allow objects to be detected by long range surveillance via their emitted or reflected heat energy [2]. To reduce the thermal signature of these objects, either the temperature of the object or its emissivity could be reduced [2], or it could be adapted to emit in the frequency between 5 and 8 μm which is blocked by the atmosphere. Textile fabrics have emissivities ranging from 0.92 to 0.96, while metallic objects such as stainless steel or aluminum have emissivities of 0.12 and 0.04–0.09, respectively [2]. The emissivity of textile fabrics can therefore be reduced by incorporating a shiny or metallic reflective cover, although this may have implications for visual camouflage [2]. For instance, Zhao and Fan coated electrospun nylon 6 nano-fibrous membranes with silver polyhedrons [41]. The authors found significant reductions in IR transmittance of the silver coated membranes, with further reductions with increased coating time.

**Figure 4:**
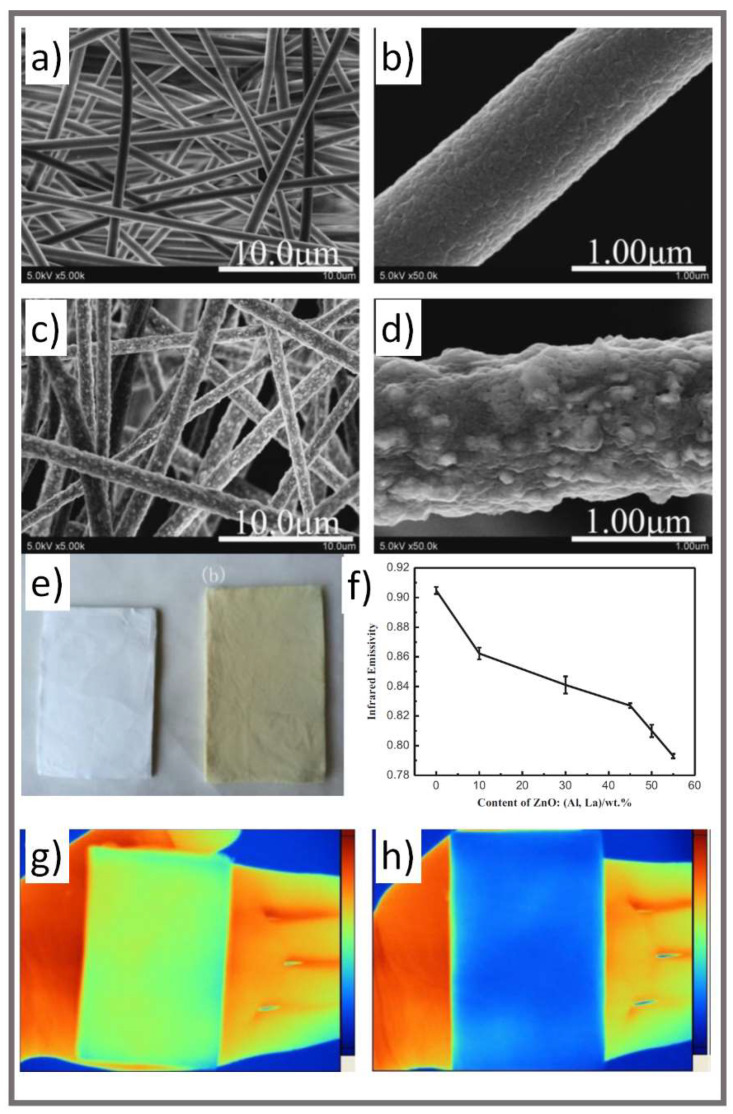
(**a**,**b**) Field emission scanning electron microscope (FE-SEM) images of electrospun pure PAN nonwoven fabrics. (**c**,**d**) FE-SEM images of electrospun ZnO:(Al, La)/PAN nanocomposite nonwoven fabrics. (**e**) Images of pure PAN and nanocomposite electrospun nonwoven fabrics, respectively. (**f**) Change in the IR emissivity vs. the nanoparticle loading. (**g**,**h**) IR images of the pure PAN and 55 wt.% ZnO:(Al, La)/PAN nonwoven fabrics, respectively (reprinted from [42] with permission from Elsevier).

Similarly, Fang et al. prepared a low IR emissive nonwoven fabric using an electrospinning process [42]. The spinning solution was composed of polyacrylonitrile (PAN) mixed with a zinc oxide:[aluminum, lanthanum] (ZnO:[Al, La]) nanoparticle powder dispersion. The IR emissivity of the nonwoven fabric decreased when the ZnO:[Al, La] powder content increased; a ZnO:[Al, La] weight ratio of 55 wt.% reduced the emissivity in the 8–14 μm wavebands to as low as 0.793 (Figure 4). At this emissivity, the nonwoven textile could match surroundings consisting of rock or withered grass outside of daylight hours (e.g., no sunshine). 

Rubeziene et al. used metallic coatings and fibers to investigate the reduction in the thermal emissivity of five fabric samples compared to a reference sample (100% polyester plain woven fabric) [43]. The treated fabric samples were as follows:

- No. 1—65% polyester/35% cotton rip-stop woven fabric, aluminum foil coated
- No. 2—100% polyester plain woven fabric, conductive metal fiber (99.6% polyester with 0.4% stainless steel staples (INOX)) inserted in weft (horizontal) direction
- No. 3—100% polyester plain woven fabric, conductive metal fiber (90% polyester with 10% INOX) inserted in weft direction
- No. 4—100% polyester plain woven fabric, with Silverflex-170^®^ yarns (98.5% polyester with 1.5% silver plated filaments) inserted in both the warp (vertical) and weft directions
- No. 5—100% polyester plain woven fabric, with Silverflex-170^®^ yarns (97.5% polyester with 3.5% silver plated filaments) inserted in both the warp and weft directions

The samples covered a heated metal plate attached to a stand with an integrated heating controller which emitted thermal energy in a manner similar to the human body. Thermal images and temperature measurements were taken of the treated samples with one, two, or three layers covering the metal plate. Compared to the standard sample, the greatest concealing effect was achieved by sample No. 1, reducing the apparent temperature over 30%; however, the rigidity and reflectivity of the sample means that it would not be appropriate for garments or concealment in the visible and NIR regions. The apparent temperature of the samples with conductive yarns was reduced by approximately 10%, with the samples containing silver having greater reducing effects than those with stainless steel due to the lower emissivity of silver.

### 2.5. Phase Change Materials

Phase change materials (PCM) refer to materials that can change their physical state from a solid to liquid, liquid to gas, or vice versa [11]. PCM are useful for camouflage applications as they can reduce fabric temperatures and, therefore, the infrared emissivity of objects. PCMs can be encapsulated into micro or macro polymer shells so that the material is contained inside during the phase change [20], while the outer shell remains solid [44]. Encapsulated PCMs are then either applied to the surface of a textile as a coating [45] or added to a polymer matrix and wet spun into filament fibers to be woven or knitted into a textile [20,46]. Solid–solid PCMs with a reversible change between amorphous, semi-crystalline and crystalline phases have also been developed [47]. Solid–solid PCMs have the advantage of small volume changes and are leakage-free during phase changes [47,48], thus avoiding the need for microencapsulation [20].

**Figure 5:**
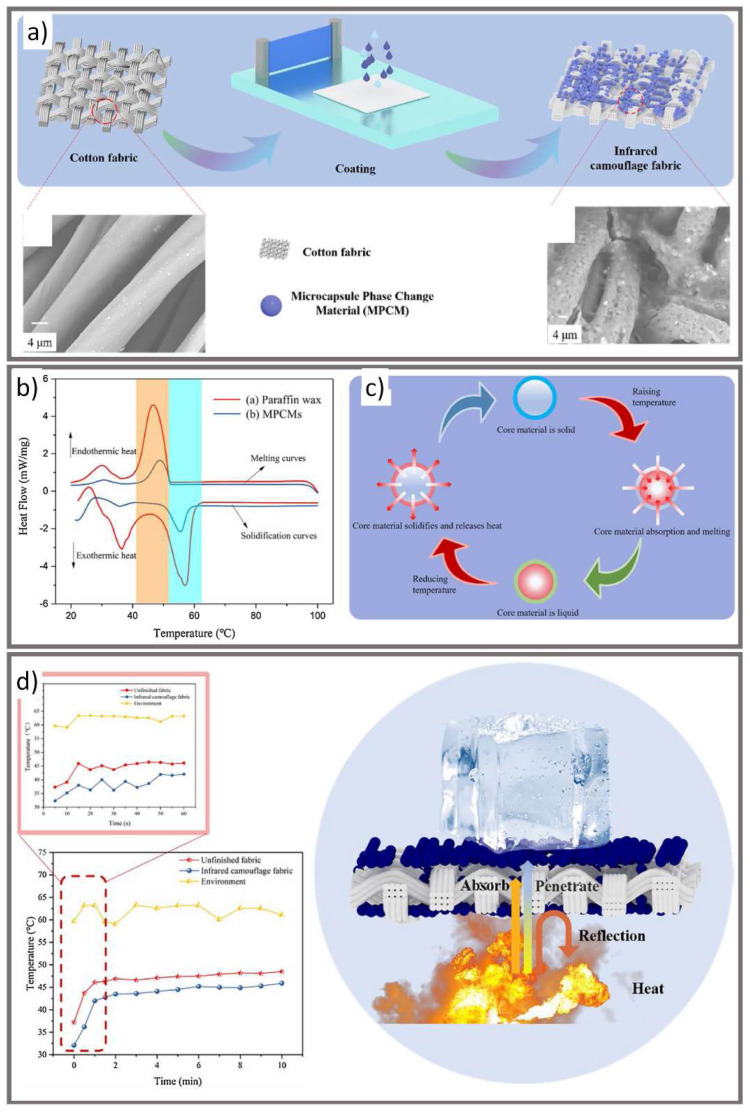
(**a**) Schematic illustration of the fabrication of IR camouflage fabric. (**b**) Differential calorimetry scanner (DSC) curves of microcapsule phase change material (MPCM) and paraffin wax. (**c**) The working principle of MPCMs. When heat is applied to the MPCMs, the core material absorbs the heat and thus melts and, when it cools again, it releases the heat and solidifies. (**d**) Temperature test curve of the unfinished versus IR camouflage fabric at 60 °C (left) and an image demonstrating the IR fabric’s temperature resistance (right) (reprinted from [44], with permission from Elsevier).

Xu et al. microencapsulated paraffin wax into urea-formaldehyde resin shells via in situ polymerization (Figure 5) [44]. These phase change microcapsules were then dispersed on both sides of a cotton fabric along with additives and thickener before being dried and cured. The infrared camouflage fabric had a lower emissivity compared to human skin and the untreated cotton fabric and a surface temperature 5–10 °C cooler than the untreated fabric when heated to 60 °C. At increased temperatures, the phase change microcapsules absorb heat, changing from solid to liquid, and do not allow the heat to pass through to the other side of the fabric, thus keeping the overall temperature of the wearer down. The authors also tested the washability, flexibility, and fastness of the coated fabric, finding it still had good infrared camouflage ability after these tests. 

Paraffin wax microcapsules were also developed in a one-pot method by Liu et al., this time by embedding the wax in a graphene oxide (GO) platelet-patched shell structure (PDVB) for improved thermal conductivity [49]. In an experiment, they coated different composite films with increasing phase change temperatures (PCT) onto four metal sample stages set up in a series circuit and heated with a resistance wire. The composite films were pure poly(dimethylsiloxane) (PDMS); octadecane@PDVB@GO@PDMS (PCT at 28 °C); eicosane@PDVB@GO@PDMS (PCT at 35 °C); and octacosane@PDVB@GO@PDMS (PCT at 68 °C). They found that the composite films with higher PCTs compared to the ambient temperature had good infrared false detection while the films with PCTs close to the ambient temperature had a good infrared stealth effect. While the microcapsules were not coated on a fabric surface, the authors suggest their one-pot encapsulation method would be suitable for other core PCMs to achieve the required temperatures for other applications such as active thermal camouflage and stealth.

### 2.6. Shape Memory Materials

Shape memory fibers have the benefit of having sensing and actuating properties within a single material [11]. These fibers are set at a high temperature using a physical restraint to shape them into a “permanent” form and can be set in a second, temporary shape when the temperature is reduced [11] (p. 48). Upon reheating, the fibers “remember” the permanent form and resume the original shape. Shape memory polymers (SMP), which typically contain networks of two polymers with different glass transition or melting transition temperatures, can be similarly set by first melting and extruding the polymer with the higher transition temperature, where it is set into a permanent shape and cooled [11]. The material is then heated above the transition temperature of the second polymer and then formed into a temporary shape. Again, when the polymer is reheated to the higher transition temperature, it reforms to the original shape. 

**Figure 6:**
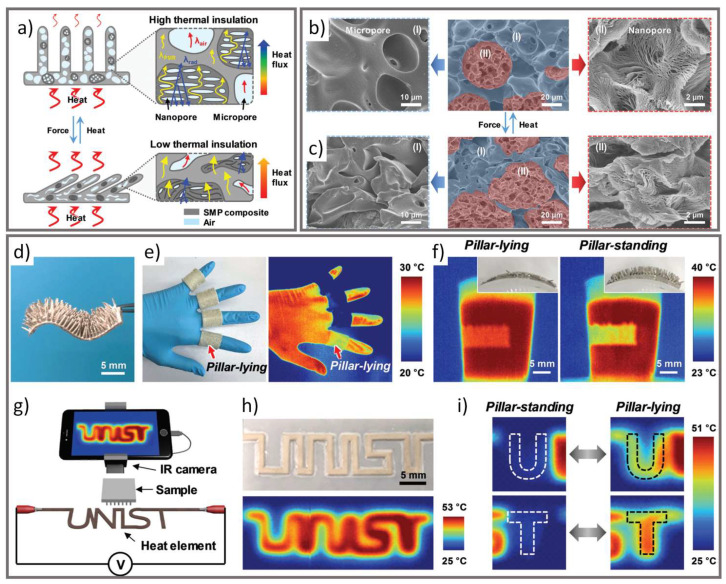
(**a**) Schematic illustration of hairy SMP structure before and after deformation and its effect on the thermal insulation property. (**b**,**c**) Cross-sectional scanning electron microscope (SEM) images of micropores and nanopores of original and deformed SMP membrane. (**d**–**f**) The effect of SMP hair standing (pillar-standing) and deformed (pillar-lying) structures on thermal insulation property. (**g**–**i**) IR images of thermal encrypted texts and other letters (republished from [50] with permission of Royal Society of Chemistry; permission conveyed through Copyright Clearance Center, Inc.).

Polyurethane is often used as a SMP because its transition temperature is easily controllable [11]. In their study, Choe et al. developed a thermally actuated SMP composed of thermoplastic polyurethane and polylactic acid with micro/nanoporous structures inspired by the hairy skin of homeothermic animals (Figure 6) [50]. The SMP resembles skin with “hair” that stands in response to temperature changes, with the polymer shape reconfiguring between hair- or pillar-standing and pillar-lying states. In the pillar-standing state, the shape memory polymer has greater thermal insulation due to the disturbed heat transfer of the standing hairs and porous structure. In addition to the thermal insulation, the authors demonstrated the potential IR camouflage of the hairy shape memory polymer with areas of the polymer becoming invisible in the IR region when in the pillar-standing state.

### 2.7. Actuation Strategies

In addition to the thermally actuated solutions described in previous sections, different mechanical actuation technologies can be envisioned for the smart camouflage textile. For instance, Suzumori et al. demonstrated the use of pneumatic rubber actuators to move a manta type robot underwater by placing the actuators in the fins of the robot [51]. These actuators consist of a reinforced fiber rubber structure with internal chambers. When pressure is applied to one chamber, the actuator bends in the opposite way, driving the robot forward.

Artificial muscles refer to devices or materials that can expand, contract, or rotate within a single body when an external stimulus is applied [52]. Artificial muscles, also known as intelligent actuators due to their sensing, processing, and actuation capabilities, have been proposed to functionalize polymer materials into smart textiles [53]. Polymer gel artificial muscles, for instance, can be chemically (e.g., pH change; solvent exchange; oxidation/reduction; ion strength change) or physically actuated through light, temperature, physical deformation, or electric or magnetic field application which can cause swelling and de-swelling of the polymer gel [53]. Organic conducting polymers such as polypyrrole, polyaniline, polythiophene and poly(3,4-ethylenedioxythiophene) can also induce movement (i.e., expand or contract) or generate forces by the incorporation or expulsion of ions [54]. Fibers with a highly oriented polymer structure such as nylon have been demonstrated as a thermally actuated artificial muscle in Mirvakili et al.’s study [55]. Nylon monofilaments were twisted into a coil structure and silver paint was applied during twisting. When Joule heating was applied, the artificial muscle achieved up to 29% tensile actuation, demonstrating a simple and easily accessible technology made from common materials such as a nylon fishing line or sewing thread. 

**Figure 7:**
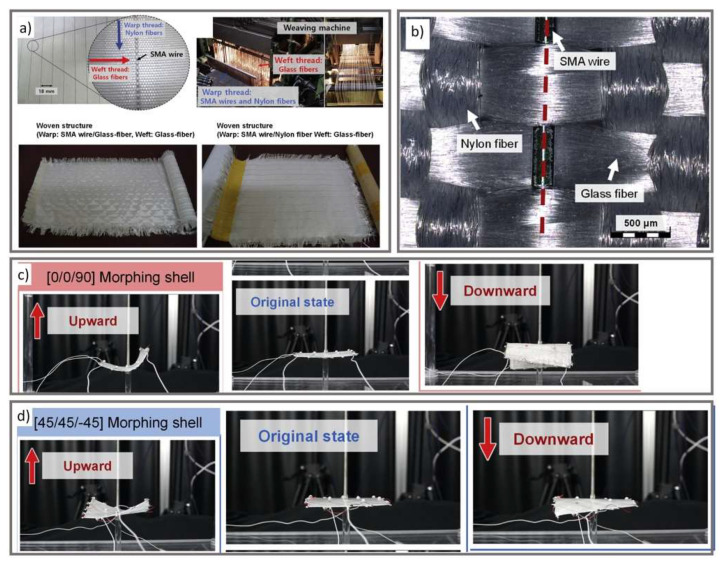
(**a**) The fabrication process of shape memory alloy (SMA) textile containing SMA wires, nylon, and glass fibers. (**b**) A closer look at the SMA woven textile. (**c**,**d**) Pictures of a morphing shape memory textile composite prepared with [0/0/90] and [45/45/−45] laminates in the original state and well as in upward and downward positions (reprinted from [57] with permission from Elsevier).

As with artificial muscles, morphing materials can autonomously change shape under specific environmental conditions [56]. Shape memory alloys (SMA) and piezoelectric materials have been used as morphing mechanisms for various engineering applications [56,57]. In their review, Wang et al. highlighted several variable rigidity materials, including shape memory polymers, that can reversibly switch from a load bearing, rigid state to a flexible, compliant state when tuned using different stimuli [58]. Han et al. developed shape memory textile composites to create a soft morphing shell capable of deforming in an upward and downward motion (Figure 7) [57]. The shell consisted of three layers of woven textile sheets embedded in a PDMS polymer matrix. The sheets were woven with nylon and glass fibers as well as SMA wires. When a current is applied to the SMA wires, the shell can deform at various angles.

## 3. Current Progress on Adaptive Camouflage

**Figure 8:**
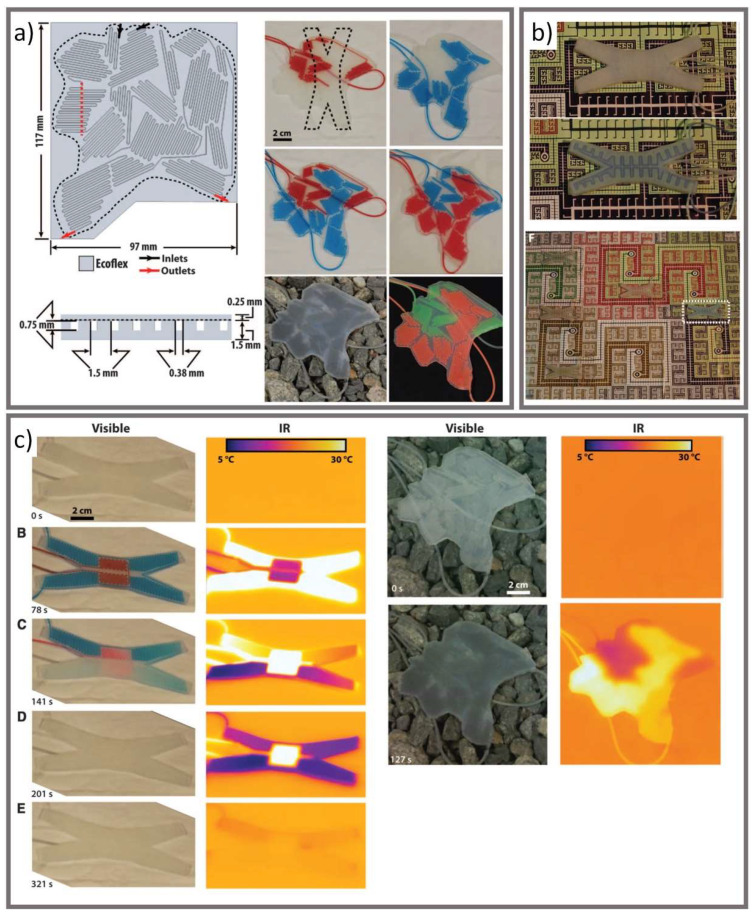
(**a**) The design of a soft robot device with internal microfluidic patterns. (**b**) The device can change its apparent color through pumping a dye solution or colored pigment that matches the environment making it invisible to the naked eye, (**c**) or can change its IR appearance through pumping warm and cool liquid (republished from [60] with permission of American Association for the Advancement of Science; permission conveyed through Copyright Clearance Center, Inc.).

Biomimicry has been applied to textiles for centuries as humans attempt to imitate the unique properties of natural life [59]. For camouflage textiles, inspiration has been drawn from animals with active camouflage capabilities such as soft-bodied cephalopods (e.g., squid, octopus, cuttlefish), which can both change their color and counter illumination [59]. Morin et al. developed a soft machine (i.e., a machine made of flexible reinforcing sheets and soft polymers) capable of changing its color, pattern, apparent shape, contrast, luminescence, and surface temperature using thin, silicone elastomer sheets (Ecoflex) with embedded microfluidics (Figure 8) [60]. The microfluidic channels are pumped with temperature-controlled or colored fluids through pneumatic pressurization to simultaneously change the visual and IR appearance of the soft machine. In one of their demonstrations, Morin et al. pumped cool (2 °C) and warm (70 °C) colored solutions through the micro channels to create contrasting patterns in the IR spectrum [60]. While this solution may not be directly suitable for garment applications due to the need to carry pumps or fluids [19,60], it demonstrates how microfluidic designs to alter IR appearance could be a worthwhile avenue for further research in military textile applications.

Yu et al. developed a multilayer unit system to imitate the coordinated actions of cephalopod skin layers [61]. These layers, from top to bottom, included: (1) a transparent photopatternable polymer matrix with embedded thermochromic dye microcapsules to mimic the rapid switching of chromatophore organs expanding or retracting; (2) a thin layer of silver imitating leucophores which provide a white background to contrast the pattern on top; (3) a silicon diode actuating layer providing Joule heating to control the thermochromic dye’s optical properties, effectively acting as an artificial muscle; (4) a layer of polydimethylsiloxane (PDMS) for mechanical flexibility; and (5) a silicon photodiode and blocking diode providing multiplexed photodetection. The layered units were configured in a 16 × 16 array to form a flexible skin. In their experimental procedure to demonstrate the system’s camouflage function, the 16 x 16 array was placed on a black-and-white patterned background. The white portion of the background was created by light passing from below through an amplitude mask. When an associated photodetector threshold was exceeded, signals from externally controlled electronics were sent to the actuators, thus changing the individual units white to mimic the background pattern.

**Figure 9:**
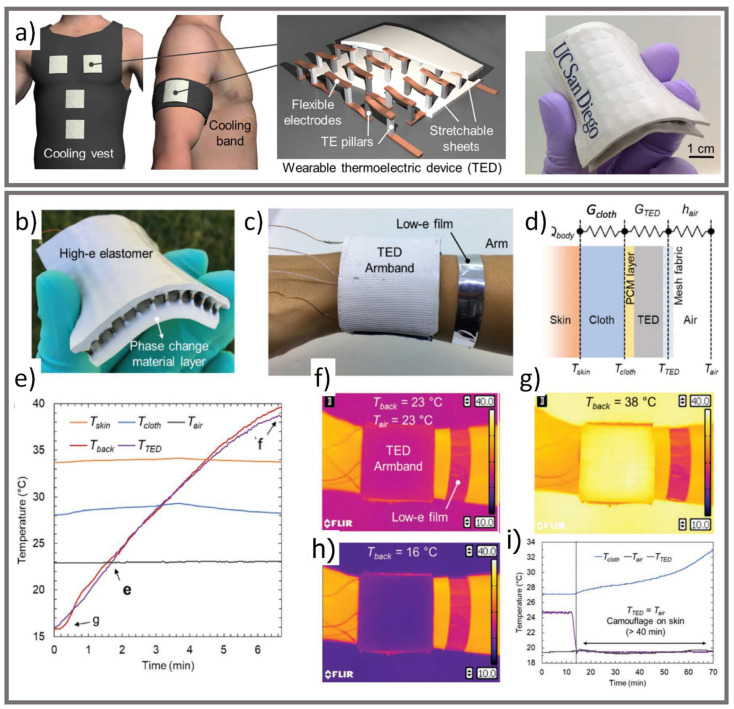
(**a**) Schematic of cooling vest with wearable TEDs patches (reproduced from [62], under CC BY-NC 4.0). (**b**) Flexible PCM/TED, (**c**) worn as an arm band, (**d**) corresponding thermal resistance circuit, (**e**) variation of the temperature as a function of time, (**f**–**h**) IR images of the band recorded at 23 °C, 38 °C, and 16 °C, respectively, (**i**) camouflage effect of the TED on the skin over time (reproduced from [19] with permission from John Wiley and Sons).

On the other hand, active thermal camouflage approaches can be divided into two main categories [19]: (1) controlling the IR emissivity or reflectivity of an object, or (2) a thermotics approach whereby materials with non-uniform thermal conduction or refractive index distribution are structurally designed to guide thermal radiation and heat conduction and help to shield the material’s thermal signature. Low emissivity approaches are limited to a certain temperature range (e.g., the object temperature must be higher than the background temperature), they can unintentionally reflect the thermal signature of surrounding objects, and they can be compromised by dust or moisture [19]. The thermotic approach has been demonstrated by hot/cold liquid injection into soft robotics [60] as well as thermoelectric devices (TED) added to rigid vehicles [19]; however, these techniques are not suitable for garment applications due to their required bulk, rigidity, and related heat sinks [19]. In response to these issues, Hong et al. proposed a flexible and wearable TED [19,62]. Their device was composed of TE pillars connected by copper sheets embedded in between two stretchable, elastomer sheets made of Ecoflex incorporated with aluminum nitride particles to improve temperature uniformity (Figure 9). The TED was covered with a high emissivity elastomer polymer layer to reduce reflectivity. Finally, in their experiments with human skin, a PCM layer (melting point of 28 °C) was added between the TED and the fabric in contact with the skin to act as a heat sink and accommodate for Peltier heating and cooling. The device demonstrated good camouflage properties in that it could match the background temperature between 16–38 °C and could provide thermal comfort for the wearer.

Xiao et al. developed a thermal camouflage system composed of vanadium dioxide (VO_2_), carbon nanotube, and graphene (VCG) thin films layered in a sandwich-like configuration [63]. VO_2_ is an ideal material for adaptive thermal camouflage as it functions as a thermochromic material, transitioning from insulator to metal around 68 °C (340 K; [63,64,65]). The VCG film (5 mm x 5 mm) experienced a complete phase transition with a small power supply of 10 mA (approximately 4.2 mW/mm^2^). In addition, the VCG film had a large tunable emissivity (∼0.86 at 40 °C and ∼0.49 at 90 °C) due to the efficient conductivity of the graphene/carbon nanotube substrate which drove the VO_2_ phase transition. To demonstrate the adaptive thermal camouflage properties of the system, the VCG film was attached to a background substrate of electrical insulation tape and connected to copper foil electrodes coated with silver thermal glue. The film was heated by an electrical source meter and thermal images of the film taken. The authors found that the VCG film thermal radiance could be electrically radiated to match the background substrate. Their adaptive camouflage free-standing film can be applied to any flexible substrate, including textiles, as a thermal cloaking application.

**Figure 10:**
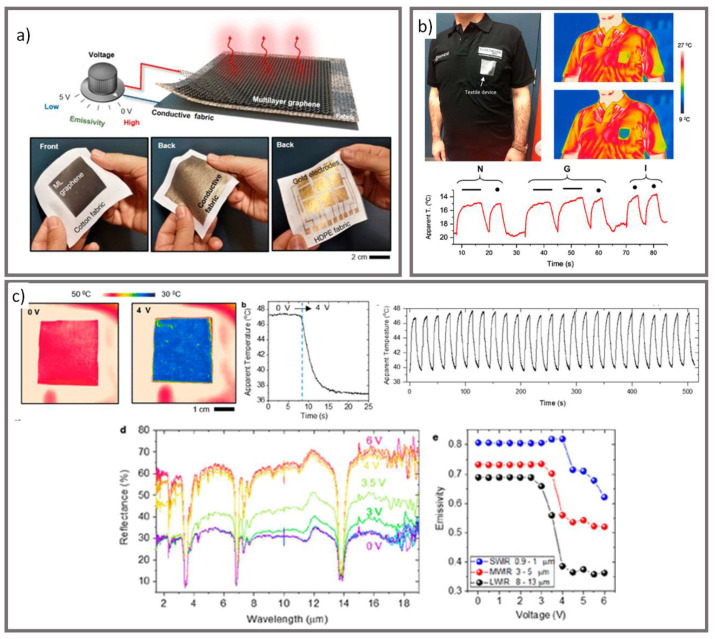
(**a**) Schematic of the adaptive IR textile consisting of multilayers of graphene, fabric, and back gold electrode layer. (**b**) IR communication t-shirt. By changing the emissivity of the patch on the t-shirt with a specific modulation, letters can be sent in morse code. (**c**) IR images of the ion intercalation adaptive optical textile in the high (0 V) and low (4 V) emissivity states while on a hot plate at 55 °C, change in apparent temperature after application of a 4 V voltage, change in apparent temperature under repeated voltage cycles, IR reflection spectra for different applied voltages and short-, medium-, and long-wavelength IR emissivity as a function of the applied voltage (reprinted from [66] with permission from American Chemical Society).

Recently, Ergoktas et al. demonstrated an adaptive optical textile with IR and NIR emissivity and reflectivity controlled via reversible ion intercalation (Figure 10) [66]. The multi-layered textile was composed of a laminated, IR transparent polyethylene film, chemical vapor deposition-grown multilayer graphene, fabric separator (woven cotton fabric), and electrode layer of conductive fabric. An ionic liquid electrolyte was then applied to the electrode layer and diffused into the fabric separator layer. When a voltage difference (>2.5 V) is applied to the multilayer graphene and electrode layer, the ionic liquid intercalates into the multilayer graphene, which functions to conceal the temperature of the device by enhancing optical conductivity and suppressing emissivity. A potential negative of this multi-layer textile if developed into a full body garment would be the added weight and need for additional power supply.

## 4. Discussion and Path Forward

The opportunities for incorporating microstructures into textiles for the purpose of visible and infrared camouflage are promising. The above review demonstrates how research is already combining various technologies into single smart textile systems to achieve more effective camouflage properties, for instance at the fiber stage (e.g., embedded additives), in woven or knitted structures, and as surface finishes, coatings, or laminations. However, it is important to keep in mind the challenges in balancing the addition of smart microfabrication with the functional requirements of textiles. Several factors must be considered when it comes to incorporating smart technologies into garments to be produced at industry scale and in a cost-effective manner. These considerations are outlined in this section along with possible strategies for improved visible and IR signature management. 

### 4.1. Functional Requirements of Smart Textile Systems for Camouflage Applications

Synthetic fibers (e.g., polypropylene, polyethylene, polyester, nylon) are well suited for smart textile manufacturing as their properties can be modified during the production process [4]. Such modifications include changing fiber size and shape, the fiber’s molecular structure, embedding additives into a polymer, or co-extruding multiple polymers at once [4]. While durable and having high tensile strength, synthetic fibers can be less comfortable as they have decreased moisture absorbency compared to natural fibers [4]. Absorbency and air permeability can be further diminished with added finishes, coatings, or lamination [4]. Therefore, systems must not add to the thermal physiological stress of the wearer, especially if the wearer is already carrying heavy gear and performing physical tasks, as is the case with many military applications [2]. 

The work of many researchers demonstrates the importance of wearer comfort, resistance to laundering, and maintaining the functionality of the garment over time [14,16,23,31,34,39,40,44]. There are benefits in avoiding the need for embedded electronics as this adds complexity in terms of washing and powering of devices, especially since energy harvesting capabilities are not ready for use in textile systems yet. Similarly, the compatibility, modularity, interoperability, and ergonomics of the smart textile system with multiple components should be factored in through intuitive design [8], user-centered design, wear trials, and testing to adhere to existing textile standard specifications. 

Furthermore, smart textile systems must consider the current manufacturing capabilities in the textile industry as some camouflage methods are expensive (e.g., the use of expensive materials or processes) and not suitable for industrial manufacturing (e.g., relying on processes not easily scalable or the use of toxic compounds) [33]. The integration of metal fibers and wires or metallic and galvanic coatings must be carefully considered as these can cause damage to textile machinery, add weight to a textile, or be limited due to adhesion difficulties and corrosion resistance [9]. Technologies can certainly be combined to develop minimum viable products, but consideration of who will pay for the products must be taken into account. Smart microfabrication should take advantage of existing infrastructure and manufacturing processes to minimize costs and consider the needs and usefulness (e.g., comfort, protection) of these products for industry and end-users. 

Smart textiles combine materials from different sectors such as textiles, electronics, and chemicals, making standardized testing a particular challenge [8]. Smart textiles for military applications must also consider adherence to military textile standards. As of December 2020, only eighteen standards exist or are in development for smart textiles, indicating a current lack of standardization, comparability, and quality-control of smart textile products [67]. As smart textiles are worn close to the body, health and safety risks, durability, and compatibility with other materials should be considered. Shuvo et al. suggests a tri-factor framework for assessing smart textile performance which includes the durability, safety, and efficiency of the product, as well as the product features and longevity, user experience, and the cost versus benefits of the product [67]. As military personnel face a multitude of threats, compromises between protection, comfort, and the ability to complete tasks while wearing protective clothing must be made [6]. However, this protection may only be required for a short amount of time during the overall wear period [8]. Solutions that are adaptive or only “on” when needed can help to balance protection and comfort [8], thus minimizing these trade-offs.

### 4.2. Three-Dimensional Printing with Metamaterials and Microfluidics

A response to these different requirements may be found by taking advantage of the opportunities offered by three-dimensional (3D) printing to produce fabric structures combining metamaterials and microfluidics. For instance, polymer-based nanophotonic was used to produce a hybrid metamaterial radiative cooling textile [68]. An electrospun layer of Si_3_N_4_ nanoparticle/poly(vinylidene fluoride) nanocomposite nanofibrous mat was sandwiched between a nanoporous polyethylene layer on the outer side and a dopamine-modified nanoporous polyethylene layer on the inner side. The resulting fabric combines high spectral selectivity, IR absorbance/emittance, and sunlight reflectance. It is also water-tight and water vapor permeable. Another example is a 3D metamaterial absorber textile structure that was proposed for radar stealth application [69]. It combines a copper yarn weft-knitted fabric as the periodic resonator on one side, a conductive plain weave fabric on the other side, and a silicone dielectric layer in the middle. Using the Computer Simulation Technology software program, the authors calculated an absorption between 81 and 95% in the 8 to 12 GHz frequency range. The use of hierarchical metamaterials allows designing solutions for multispectral camouflage, i.e., covering both infrared and microwaves [70]. This was achieved by integrating an IR selective emitter with a microwave selective absorber. The authors managed to reduce the signature levels of 8–12 μm IR waves by up to 95% and 2.5–3.8 cm microwaves by up to 99%.

Three-dimensional printing has opened up new perspectives for manufacturing metamaterials. In particular, it has been shown to allow the production of microstructures capable of experiencing large deformations at the microscale using embedded soft pivots [71]. For instance, 3D printed pantographic sheets comprised of straight and parabolic fibers connected by soft pivots were subjected to bias extension up to rupture. The stress–strain curves were successfully described using continuum bidimensional models. Other deformable metamaterial complex structures produced by 3D printing include stretchable circuits manufactured by coextrusion of liquid metal within thermoplastic filaments [72] and a robotic gripper combining soft and hard thermoplastics [73]. Recent progress in 3D printing includes the use of a polycarbonate (PC)/acrylonitrile butadiene styrene (ABS) core/sheet filament as feedstock for fused filament fabrication 3D printing, yielding a ductile and tough composite ABS/PC meso-structured part after annealing at a temperature between the glass transition temperatures of ABS and PC [74]. Such techniques could be adapted for more than just mechanical improvements and open up opportunities for complex internal structures of fibers to be manufactured with future printing and thermal drawing steps. 

In another area, droplet microfluidics can be used to form specialized microparticles with well controlled and tunable size, structure, and composition [75]. After the droplets are formed one by one using breakout methods such as dripping, jetting, or squeezing, they can be converted into solid microparticles by polymerization, temperature-induced gelation, ionic crosslinking, or solvent evaporation for instance. The microfluidic devices most commonly used to generate and manipulate droplets are glass capillary microfluidics and poly(dimethylsiloxane) (PDMS) devices produced by lithography or molding processes [75]. Recently, 3D printing has been explored with some success for the fabrication of microfluidic devices. However, some challenges remain, including the needed resolution, suitable materials, and surface modification techniques. If these precisely engineered microparticles have found numerous applications in medicine, for example for drug delivery and cell encapsulation [75], they can also be used to provide solutions for adaptive camouflage by combining chromatophores with hyperelastic matrices and varying their in-plane surface area by compressing them in the other direction. 

**Figure 11:**
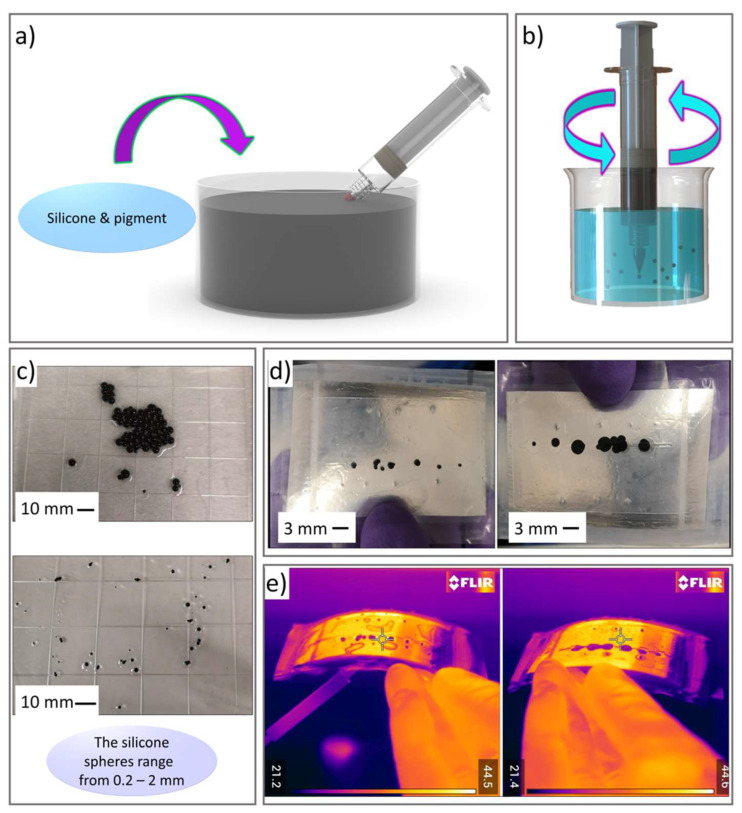
(**a**,**b**) Schematic illustration of the fabrication of silicone based chromatophores spheres. (**c**) Sizes of the silicone spheres that can be achieved depending on the speed of stirring and the gauge of the dispensing needle tip. (**d**) The silicone spheres after placement in an IR transparent polyethylene pouch with a metalized background. (**e**) Images of deformed and undeformed spheres using a FLIR IR camera while reflecting a warm object to demonstrate an apparent local change in temperature.

Figure 11 demonstrates the fabrication of one such microfluidic device developed by the authors using silicone-based chromatophore spheres. In this process, Ecoflex gel was mixed with silicone fluid (ratio of 1:2) and various pigments to aid in visual identification. After degassing, a disposable syringe was used to collect the silicone material and inject it while being stirred in warm (~70 °C) soapy water which ensured the emulsion remained stable long enough for the silicone to cure (~5 minutes). The size of spheres could be controlled by the stirring speed and the gauge size of the dispensing needle tip, with different sieves used for collection producing spheres in the range of 0.2–2 mm. The silicone spheres were then placed in an IR transparent polyethylene pouch with a metalized background. By applying vacuum to the pouch, the spheres were deformed and covered the low emissivity surface. Placing greater numbers of spheres in controlled spots of the pouch has the potential to change the entire surface of the device from IR transparent to IR opaque. Through applying nearly 20 years of research in droplet microfluidic technology towards the manipulation of thermoplastics, liquid metals, gels and curable polymers, entirely new fabrics made of microfluidic channels/capillaries may be manufactured for variable thermal and visible properties for a variety of applications. Applying microfluidic techniques to the production of smart fabrics is very early in its development, however, and challenges in the adoption of these technologies by large manufacturers to make economically viable products, as well as investigations into durability, adaptability and comfort as fabrics, remain an open research area for these new smart materials. 

### 4.3. Machine Learning

Machine learning for control over manufacturing and process stability of complex, multi-material extrusion and thermal drawing may also help alleviate the current challenges experienced when attempting to apply current technologies for the development of smart visible and IR camouflage solutions, especially when considering the complexity of combining several techniques in a single device. The complexity of controlling non-equilibrium processes with highly variable viscosity, viscoelasticity, shear thinning behavior and surface tension-induced droplet breakups balancing with multi-material extrusion leads to extreme difficulties in predicting all final part behavior with simple input conditions. Machine learning has been successfully used to improve the performance of textiles, for instance with the optimization of the elastic modulus of woven fabrics based on the weave factor, warp yarn count and pick density using artificial neural network (ARN) and random forest regression (RFR) approaches [76]. In an attempt to solve the challenges encountered with asymmetric interactions in thermal metamaterials, an autoencoder was also trained to optimize thermal transparency using two types of particles disposed in a periodic manner in a mechanism named periodic interparticle interaction by the authors [77]. It allowed for using a more complex lattice and varying the relative positioning of the two types of particles. A vast array of data on multi-material extrusions would need to be collected to train appropriate machine learning algorithms, but once completed the improved predictive performance of new material combinations, processing conditions and post-processing treatments for smart fibers/fabrics could unlock new capabilities for intelligent and responsive fabrics. 

### 4.4. Interdisciplinary Approaches 

Finally, the most critical aspect towards achieving comfortable and durable smart camouflage textiles that can be manufactured in a cost-effective manner is a holistic approach involving the different relevant disciplines: textiles and clothing, materials, design, engineering, and manufacturing. An interdisciplinary perspective that considers the needs of the users and the capability of the manufacturing industry at the initial step of the development is key to solving this multifaceted problem involving dynamic interactions between humans and their changing environment. Researchers must work closely with textile manufacturers to ensure that the developed technologies will be feasible and scalable, applying cutting edge approaches from non-traditional disciplines to textile design, but also understanding early in the process the commercial and industrial realities of large-scale manufacturing systems through frequent dialogue. In this context, an innovation-oriented industry aiming at high value-added niche products and capable of production flexibility and fast adaptation such as the textile industry is an ideal partner for such endeavor. 

**Figure 12:**
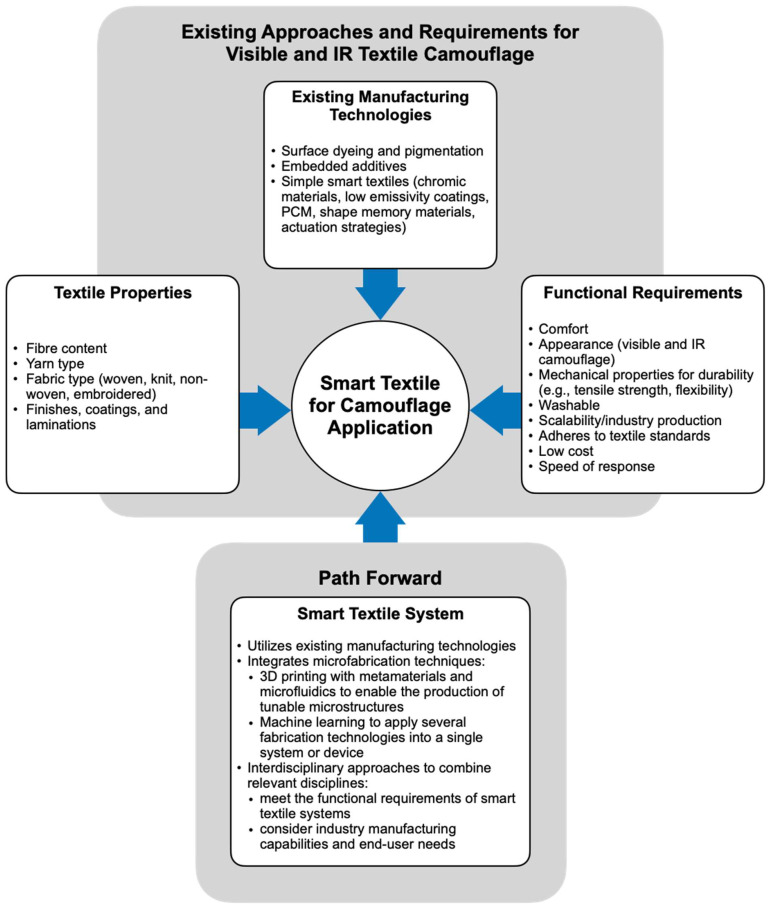
Summary of textile properties, existing manufacturing technologies, and functional requirements for visible and IR smart textile camouflage and the proposed path forward for a smart textile system using existing manufacturing technologies along with microfabrication techniques and interdisciplinary approaches.

Figure 12 summarizes existing technologies used for smart textile systems for visible and IR camouflage and this proposed path forward. Smart textiles for camouflage application must consider various textile properties that will influence performance while also fulfilling functional requirements such as comfort, appearance, durability, and manufacturing scalability. As demonstrated in this review, existing manufacturing technologies with increasing smart capabilities have achieved visible and IR camouflage in textiles, but still have limitations in terms of speed of response, weight, washability, and wearability. An envisioned path forward for the field takes an interdisciplinary approach that utilizes existing manufacturing techniques and considers the diverse functional requirements of wearable textiles while integrating microfabrication techniques such as 3D printing and machine learning to develop smart textile camouflage systems. 

## 5. Conclusions

Smart textiles offer great potential for the next generation of camouflage products. While preserving the comfort and wearability of traditional textiles, they can take advantage of various existing technologies to provide the adaptive visible and IR signature management that is critically needed due to the advances in infrared sensing. These technologies, which can be combined to work in synergy, include surface coloring and pigmentation, embedded additives, chromic materials, low emissivity coatings, phase change materials, shape memory materials, and different thermal and mechanical actuation strategies. Combining these technologies can overcome some of the limitations outlined in Table 1. Current work on adaptive camouflage has looked at nature as a source of inspiration, with embedded microfluidics and microcapsules. In terms of active thermal camouflage, the strategies used generally rely on IR emissivity/reflectivity control or thermal radiation/heat conduction guiding. 

However, many challenges remain on the path towards adaptive camouflage for clothing applications. The garment must be comfortable, durable, and easy to maintain. The manufacturing process must be compatible with industry capabilities and cost effective. Therefore, solutions proposed must consider the trade-offs between function, comfort, and cost to produce camouflage smart textile systems that can be manufactured at scale. In addition, the development of appropriate test methods for quality control is critically needed. A response to these different requirements may be found by taking advantage of the opportunities offered by 3D printing to produce fabric structures combining metamaterials and microfluidics. Above all, an interdisciplinary, holistic approach that considers the needs of the users and the capability of the manufacturing industry at the initial step of the development is key to solving this multifaceted problem involving dynamic interactions between humans and their changing environment.

## Figures and Tables

**Table 1:** Advantages and limitations of applying existing technologies in smart camouflage textiles.

Technology	Advantages	Limitations
Surface dyeing and pigmentation	Low cost Existing manufacturing capabilities can be used to dye and print textiles	Difficulty of dyeing synthetic fibers [13] Does not sense, react, or adapt to external stimuli Can reduce durability, air permeability, strength, and other functional properties of the textile Can alter visual color and reflectance of textile, e.g., [14,15,16]
Embedded additives	Low cost Existing manufacturing capabilities can be used for embedding additives at the polymer stage before fiber extrusion Additives fixed within the fiber	Can alter the visual color of textiles Does not sense, react, or adapt to external stimuli
Chromic materials	Repeatable color change [9] Various stimuli to produce color change Generally high speed of response for thermochromic materials [17]	Poor wash and light fastness of thermochromic dyes [18]
Low emissivity coatings and fibers	Reduction in textile emissivity Can be woven or knitted into fabric [9] High conductivity [9]	Reflectivity [19] Added weight and cost [9] Limited adhesion and corrosion resistance [9] Metallic fibers/wires are potentially damaging to machinery [9]
Phase change materials	Reversible change in phase (e.g., solid to liquid and back) [9] Thermoregulating effects [9,20]	Change only lasts for a limited time Risk of microcapsule leakage Coated microcapsules can be rubbed off or washed away [20]
Shape memory materials	Sense and actuate within one material [11] Reconfigures between original and deformed shape [11] Ease of deformation [9] Good shape memory at low temperatures for alloys [9] Can be processed into fibers, films, and membranes [9]	Speed of response Lack of extensibility of shape memory alloys in weaving/knitting processes [9] Shape memory effects reduced when mixed with other polymers before fiber extrusion [9]
